# Deletion of TGF-β1 Increases Bacterial Clearance by Cytotoxic T Cells in a Tuberculosis Granuloma Model

**DOI:** 10.3389/fimmu.2017.01843

**Published:** 2017-12-20

**Authors:** Hayley C. Warsinske, Elsje Pienaar, Jennifer J. Linderman, Joshua T. Mattila, Denise E. Kirschner

**Affiliations:** ^1^Department of Microbiology and Immunology, University of Michigan Medical School, Ann Arbor, MI, United States; ^2^Department of Chemical Engineering, University of Michigan, Ann Arbor, MI, United States; ^3^Department of Infectious Diseases and Microbiology, University of Pittsburgh Graduate School of Public Health, Pittsburgh, PA, United States

**Keywords:** cells-T cells, cytotoxic, infections-bacterial, transforming growth factor beta, tuberculosis, pulmonary, agent-based modeling

## Abstract

*Mycobacterium tuberculosis* is the pathogenic bacterium that causes tuberculosis (TB), one of the most lethal infectious diseases in the world. The only vaccine against TB is minimally protective, and multi-drug resistant TB necessitates new therapeutics to treat infection. Developing new therapies requires a better understanding of the complex host immune response to infection, including dissecting the processes leading to formation of granulomas, the dense cellular lesions associated with TB. In this work, we pair experimental and computational modeling studies to explore cytokine regulation in the context of TB. We use our next-generation hybrid multi-scale model of granuloma formation (*GranSim*) to capture molecular, cellular, and tissue scale dynamics of granuloma formation. We identify TGF-β1 as a major inhibitor of cytotoxic T-cell effector function in granulomas. Deletion of TGF-β1 from the system results in improved bacterial clearance and lesion sterilization. We also identify a novel dichotomous regulation of cytotoxic T cells and macrophages by TGF-β1 and IL-10, respectively. These findings suggest that increasing cytotoxic T-cell effector functions may increase bacterial clearance in granulomas and highlight potential new therapeutic targets for treating TB.

## Introduction

*Mycobacterium tuberculosis* is a pathogenic bacterium and causative agent of tuberculosis (TB). Approximately 1 in 3 people are infected with *M. tuberculosis*, resulting in 1.4 million deaths in 2015, including 140,000 children (1). The only vaccine for TB is a live, attenuated *Mycobacterium bovis* strain that confers some protection against severe manifestations of pediatric TB but does not offer lasting protection. With the development and spread of multi-drug resistant TB, there is a need for new, potentially host directed, therapeutics for TB (2). Current therapeutic strategies (antibiotics) require months of multi-drug treatment and treatment failures can lead to reactivation disease, sometimes years after initial infection (3). Developing new alternative therapies to address TB will require an improved understanding of host immune responses to *M. tuberculosis*.

The most common outcome of infection is formation of dense, organized immunological structures called granulomas in lungs (4, 5). Granulomas isolate infected cells from adjacent tissue and prevent bacterial dissemination but can also make it difficult for the immune system and drugs to kill all bacteria, leading to a stalemate between the immune system and bacteria (6–8). Granulomas are complex structures that can be classified by their cellular composition, the number of bacteria present, and overall shape, with a wide spectrum of observations (9–13). Granulomas are dynamic structures that change continuously over time. Three generalized categories that can be used to describe the state of bacterial burden for a granuloma are *contained*, indicating the number of live bacteria in a granuloma has stabilized over time, *disseminating*, indicating the bacterial load in a granuloma is increasing and the infection is not well controlled, and *sterilized*, indicating all the bacteria have been killed (Table 1) (13). Identifying immunological mechanisms that differentiate sterilized, contained, and disseminating granulomas could present therapeutic targets to improve TB treatment.

**Table 1:** Categories of simulated granulomas by bacterial status.

	Description	CFU by day 200	Representative simulation snapshot
Sterilized	All bacteria have been killed	0	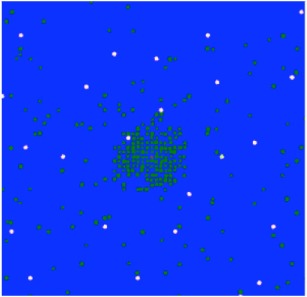

Contained	Number of live bacteria has stabilized over time	Less than or equal to twice CFU at day 100	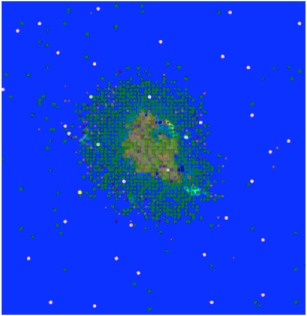

Disseminated	Number of live bacteria is increasing, infection is not well controlled	Greater than or equal to twice CFU at day 100	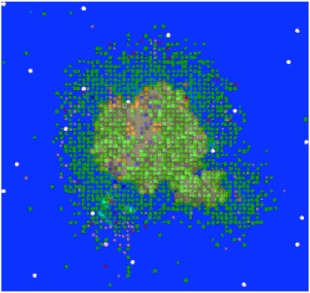

There is increasing evidence suggesting that cytokine signaling is responsible for establishing granulomas that successfully control *M. tuberculosis* infection. Pro-inflammatory cytokines including TNFα and IFNγ have been investigated for their antimicrobial functions. TNFα has been shown to induce macrophage activation (14), recruit immune cells to the site of infection by promoting chemokine secretion from macrophages (15), and can induce cellular apoptosis (16). Inhibition of TNFα during *M. tuberculosis* infection leads to unstructured granulomas in mice and increased bacterial burdens (17–19), however in non-human primates (NHPs) response to anti-TNF is different (20–22). Similarly, IFNγ is also responsible for macrophage activation during infection (23, 24). A balance of pro- and anti-inflammatory cytokines is required for establishing granulomas that successfully control *M. tuberculosis* infection (10, 18, 25, 26). In this study, we seek to characterize the role of TGF-β1, anti-inflammatory cytokine, in granuloma formation and function. A better understanding of the role of TGF-β1 in the context of TB infection could illuminate potential targets for immune therapeutics that can stimulate the host immune response to *M. tuberculosis* infection.

Anti-inflammatory cytokines including TGF-β1 and IL-10 have come under increasing scrutiny for their association with severe TB (18, 21, 27–29). TGF-β1 is highly conserved across taxa (30) and can influence many cell types (31–34) by signaling through the TGFβR1/TGFβR2 receptor complex (35). TGF-β1 has a variety of inhibitory effects including the ability to downregulate macrophage activation and effector function (36–40), decreasing cytokine secretion by macrophages and cytotoxic T cells (41, 42), and decreasing proliferation of T cells (43). Moreover, TGF-β1 inhibits effector functions in antigen-stimulated cytotoxic T cells in tumors (44, 45), and TGF-β1-expressing regulatory T cells (Tregs) suppress cytotoxic T cell function (46). TGF-β1 may also exacerbate TB by downregulating *M. tuberculosis-*specific pro-inflammatory cytokine secretion and proliferation by T cells (41, 43, 47). Systemically, *M. tuberculosis* infection upregulates TGF-β1 expression, and peripheral blood monocytes from TB patients display elevated TGF-β1 secretion (48–50). Granulomas from NHPs show high levels of TGF-β1 (51). *In vitro* studies have demonstrated that TGF-β1 promotes mycobacterial growth within mononuclear cells, and addition of exogenous TGF-β1 leads to increased *M. tuberculosis* replication (52, 53). Inhibiting TGF-β1 restricts bacterial growth *in vitro* (52, 53). Despite evidence of the effects of TGF-β1, the roles of TGF-β1 in the context of TB granulomas remain uncharacterized (51).

IL-10 is another anti-inflammatory cytokine expressed by T cells and macrophages in granulomas. It signals through its receptor, IL-10R (54), and can inhibit macrophage antimicrobial activities that are critical for protection against TB (28, 55). These actions play an important role in early granuloma formation and macrophage regulation (26, 56). *In silico* deletion of IL-10 between the time of infection and 45 days postinfection (PI) increases granuloma sterilization, and this effect is attributable to modest increases in macrophage activation (56). However, the benefit of IL-10 deletion decreases at later time points and there is an increase in potentially pathologic inflammation (56). Moreover, virulent *M. tuberculosis* strains are associated with upregulated IL-10 expression, suggesting the effects of IL-10 may have survival benefits for *M. tuberculosis* (25, 57, 58).

The effects of TGF-β1 on overall granuloma development and function, as well as the interplay between IL-10 and TGF-β1 in regulating inflammation in granulomas remain uncharacterized (18, 59). Both cytokines are elevated in the bronchoalveolar lavage fluid in patients with pulmonary TB when compared to patients with other lung diseases and healthy patients (27). These findings, and others (27, 48–50, 52, 53, 60–64), emphasize the importance of TGF-β1 and IL-10 in pulmonary TB, but do not identify their interaction during granuloma regulation (10). Previous work indicates TGF-β1 and IL-10 may differentially regulate lymphoid- and myeloid-derived cells. For example, TGF-β1 regulates lymphoid-derived NK cell involvement in T helper type 1 cell development and NK cell maturation (65), but not myeloid-derived dendritic cell involvement in T helper type 1 cell development (66). By contrast, IL-10 is a major regulator for myeloid-derived cells including dendritic cells and monocytes (67, 68). This dichotomous regulation has not been examined in TB and could significantly impact development of new vaccines and therapeutics.

With this study, we identify TGF-β1 as an important regulatory factor impacting mycobacterial control in granulomas with effects that are distinct from those of IL-10. NHPs and rabbits are the best animal models for TB with human-like granulomas, but these animals have practical limitations making their study challenging (11). We take a systems biology approach pairing *ex vivo* experimental data with computational modeling. We use an agent-based model that captures tissue, cellular, and molecular scale interactions of cells and cytokines in granulomas called *GranSim* (7, 26, 56, 69–73). *In vivo* experimental data are generated in Mtb-infected NHPs. This unique combination of computational and experimental methods represents a novel approach to investigating questions that cannot be addressed by traditional experimental systems. We find that although TGF-β1 can regulate many cell types, its regulation of cytotoxic T-cell effector function has the strongest influence on granuloma outcome. Simulated deletion of TGF-β1 in granulomas, an experiment that cannot be performed in NHPs, leads to improved bacterial clearance and lesion sterilization. We also find that the role of TGF-β1 in granulomas differs from that of IL-10, highlighting a novel differential regulation of cytotoxic T cells and macrophages. Understanding the regulatory roles of cytokines, alone and in combination, can further our ability to predict therapeutic targets.

## Materials and Methods

### Study Design

The goal of our study was to assess the role of TGF-β1 in the formation and function of a *M. tuberculosis*-induced granuloma. We used *GranSim*, a well-validated agent-based model of granuloma formation and function in the lung. We simulated granulomas in various scenarios: containment, IL-10 KO, TGF-β1 KO, IL-10/TGF-β1 double KO, IL-10 depletion, TGF-β1 depletion, and IL-10/TGF-β1 depletion. We also performed *ex vivo* studies granulomas derived from multiple cynomolgus macaques infected with *M. tuberculosis*, and analyzed them for the presence of IL-10 receptor and TGF-β1 receptor for validation of our predictions.

### *In Silico* Studies

#### Agent-Based Model

The simulation studies in this paper are performed using *GranSim*, a 2D hybrid agent-based model of granuloma formation and function in the lung following infection with *M. tuberculosis* (26, 56, 70, 71, 74–77). *GranSim* captures molecular, cellular, and tissue scale dynamics of granuloma formation. The model accounts for diffusion of chemokines and cytokines at the molecular scale (78). At the cellular scale, *GranSim* tracks individual cells, including four states of macrophages (resting, activated, infected, and chronically infected) and three distinct types of T-cells (cytotoxic, regulatory, and IFNγ producing T-cells). Interactions between cells are captured as rules in the model. At the tissue scale, *GranSim* accounts for chemokine-directed cellular movement. We include growth and death dynamics of bacteria, and account for varying growth rates between intracellular, extracellular, and hypoxic environments. Here, we coarse grain bacterial dynamics to tracking their group population dynamics, to focus specifically on the roles of key cytokines in TB. A detailed bacterial dynamic model, including response to environmental conditions, is outside the scope of this paper, and has previously been published by our group (73). Granuloma formation is an emergent behavior of the model. A complete list of model rules can be found at http://malthus.micro.med.umich.edu/GranSim. *GranSim* provides us with a large amount of information about TB granulomas. Much of this information is currently impossible to collect *in vivo*. At the molecular scale, *GranSim* predicts chemokine and cytokine concentration gradients over the entire simulation space for every time point. At the cellular scale *GranSim* tracks macrophages and T cells along with their respective states and interactions. Data derived from the model can be quantitative, e.g., expressed in numbers and concentrations over time, or it can be qualitative, e.g., in the form of snapshots that provide a spatial perspective. These snapshots can be linked to stream a time-lapse movie (http://malthus.micro.med.umich.edu/lab/movies/TGFB_GranSim/). A full list of parameters for the model is included in the supplementary material (Table S1 in Supplementary Material).

**Figure 1:**
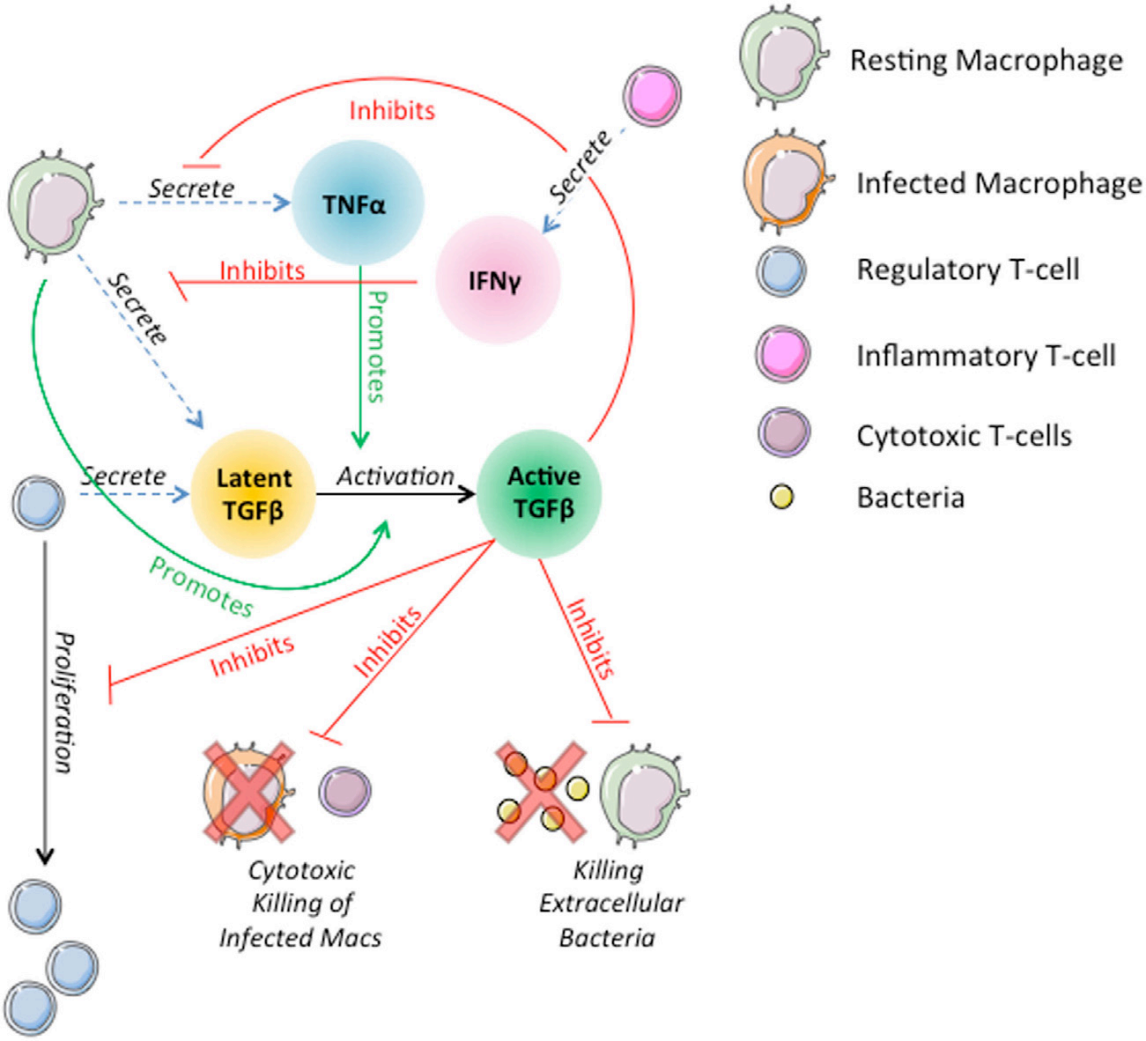
Schematic representation of physiological interactions of TGF-β1 in the hybrid multi-scale computational lung model, *GranSim*. TGF-β1 is secreted in latent form by macrophages and regulatory T-cells. Latent TGF-β1 is activated in the presence of macrophages. TNFα promotes activation of latent TGF-β1. Active TGF-β1 inhibits T-cell proliferation, cytotoxic killing of infected macrophages by cytotoxic T-cells, killing of extracellular bacteria by macrophages, and macrophage secretion of TNFα. IFNγ signaling by inflammatory T-cells inhibits macrophage secretion of TGF-β1. These interactions are included as part of the larger ABM *GranSim*. Full rules for *GranSim* are found at http://malthus.micro.med.umich.edu/GranSim.

Prior versions of *GranSim* include rules describing the actions of TNFα, IFNγ, and IL-10 on macrophages and T-cells (19, 26, 56, 71, 76, 77, 79). In this version of the model, we introduce rules governing the dynamics of TGF-β1 and its interactions with other cells and cytokines in the model (Figure 1). In the model, TGF-β1 is secreted in latent form by macrophages and regulatory T-cells. Secretion of TGF-β1 is updated on the molecular time step (Table S1 in Supplementary Material). Dynamics of latent TGF-β1 activation are represented as follows: we consider compartments that contain a macrophage to have MMP9 (80–82), which activates latent TGF-β1 within that compartment (Amount TGF β1 activated) (Table S1 in Supplementary Material). The activation of latent TGF-β1 is described by the following equation:

(1) $$\begin{array}{l} \text{Amount TGF}\beta \text{1 activated}=\\ \left({\text{Activation}}_{\text{fraction}}+\left(\left(1-{\text{Activation}}_{\text{fraction}}\right)\times \text{ TNF}\alpha_{\text{signaling}}\right)\right)\times \left[\text{TGF}\beta 1\right] \end{array}$$

The fraction of TGF-β1 that is activated is a function of TNFα concentration in the compartment where Activation_fraction_ is the fraction of TGF-β1 activated by the macrophage and (1 − Activation_fraction_) × TNFα_signaling_ is the additional amount activated in response to TNFα.

T-cell proliferation is inhibited by bound active TGF-β1 (BoundTGF-β1). The probability of proliferation is described by the following equation:

(2) $$\text{Probability of T cell proliferation}=\frac{\left(\text{TGF}\beta 1{\text{max}}_{\text{Tcell}}-\text{BoundTGF}\beta 1\right)}{\text{TGF}\beta 1{\text{max}}_{\text{Tcell}}}$$

where *TGF*β1max_Tcell_ is the amount of bound active TGF-β1 (BoundTGFβ1) that completely inhibits proliferation.

Active TGF-β1 deregulates cytotoxic T cells, inhibiting their ability to kill infected macrophages. The probability of a macrophage killing of extracellular bacteria is reduced up to 50% by active TGF-β1:

(3) $$\begin{array}{l} \text{Probability macrophage kills bacteria}=\\ \;{\text{MacKill}}_{\text{baseline}}\times \frac{\left(\text{TGF}\beta 1{\text{max}}_{\text{Mac}}-\left(0.5\times \text{BoundTGF}\beta 1\right)\right)}{\text{TGF}\beta 1{\text{max}}_{\text{Mac}}} \end{array}$$

where MacKill_baseline_ is the baseline probability a macrophage will kill extracellular bacteria and TGFβ1max_Mac_ is the amount of bound active TGF-β1 (BoundTGFβ1) that fully inhibits bacterial killing.

Macrophage secretion of TNFα is reduced up to 50% by active TGF-β1:

(4) $$\text{TNF}\alpha \text{ secretion}=\text{MacTNF}\alpha_{\text{synth}}\times \frac{\left(\text{TGF}\beta 1{\text{max}}_{\text{Mac}}-\left(0.5\times \text{BoundTGF}\beta 1\right)\right)}{\text{TGF}\beta 1{\text{max}}_{\text{Mac}}}$$

where MacTNFα_synth_ is the baseline TNFα synthesis rate and TGFβ1max_Mac_ is the amount of bound active TGF-β1 (BoundTGFβ1) that fully inhibits synthesis.

IFNγ signaling by inflammatory T cells inhibits TGF-β1 secretion by macrophages by 50% until the macrophage is no longer sensitive to the T-cell signal (Table S1 in Supplementary Material). These interactions are included as part of the larger ABM *GranSim*. Full rules for *GranSim* are found at http://malthus.micro.med.umich.edu/GranSim.

#### Simulated Granulomas and Model Calibration

**Figure 2:**
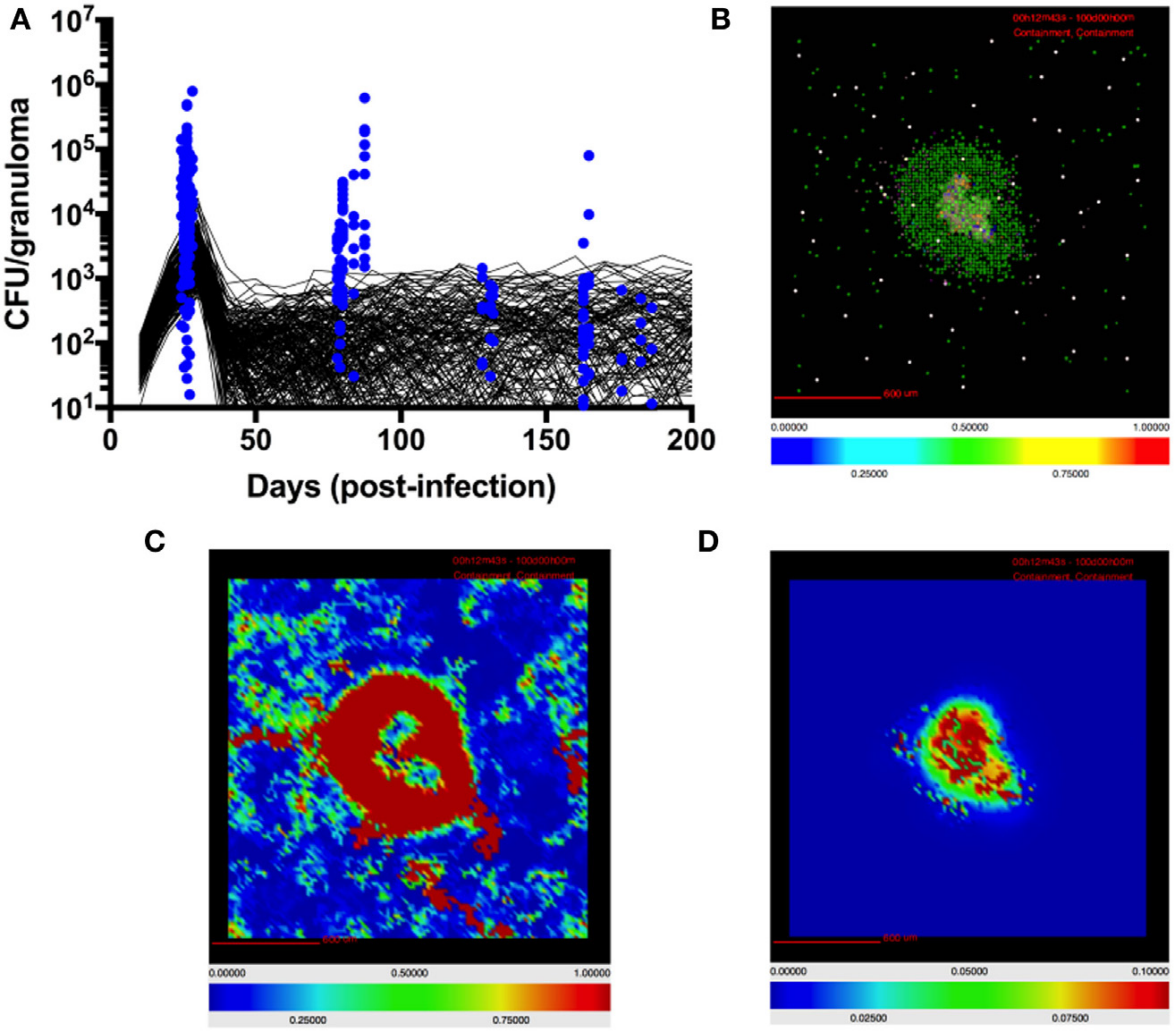
Model quantitatively and qualitatively recapitulates non-human primate (NHP) granuloma data. **(A)** CFU per granuloma of simulated granulomas scaled to 3D (see Materials and Methods) and granulomas extracted from NHPs at different time points (12, 13, 22, 83). Black lines indicate CFU/granuloma over time for 256 individual representative simulations of 1,500 total simulations. Blue dots indicate CFU/granuloma of granulomas extracted from NHPs at different time points (84). **(B)** Simulated granulomas capture spatial organization of granulomas from NHPs. Simulated granuloma showing resting macrophages (green), infected macrophages (orange), chronically infected macrophages (red), activated macrophages (blue), cytotoxic T cells (magenta), IFNγ-producing T cells (pink), and regulatory T cells (cyan) at 100 days PI. **(C)** Snapshot of simulated granuloma showing heat map of latent TGF-β1. **(D)** Snapshot of simulated granuloma showing heat map of active TGF-β1.

For this study we calibrate parameters in *GranSim*, including TGF-β1 parameters, to an NHP dataset of granulomas (Figure 2) (13, 21, 83). Bacterial load (measured in colony-forming units, CFU) per granuloma is scaled from 2D to 3D for our simulated granuloma for direct comparison with NHP data (Figure 2). Model output scaling is performed as described in prior work (56). Model calibration produces our baseline parameters, which satisfy criteria for containment as previously described in Ref. (73). Because of the stochastic nature of *GranSim*, multiple simulations are required to capture all of the dynamics. In NHPs, the median number of granulomas per infected individual is 46 (83). It has been shown that each NHP granuloma has a unique trajectory (83). To capture this naturally occurring variation, we vary the baseline parameters by 20% and simulate 500 unique granulomas each in triplicate (to account for stochastic as well as parametric uncertainty) (79). From these, we distinguish disseminating granulomas from containment granulomas. There are 1,337 granulomas that comprise our baseline containment set, and 163 granulomas that disseminate and are excluded from our baseline set. At 200 days PI, we observe about 50% of simulated granulomas sterilize their bacterial load. This is consistent with previous modeling and animal studies (56, 83). The granulomas that do not sterilize are considered contained if they have a CFU below 2,500 at day 200, and have fewer than twice as many CFU at day 200 than they did at day 100 PI. An occasional outlier in this set will progress to dissemination. We repeat this same model calibration process to obtain a parameter set representing to generate a baseline set of simulated disseminating granulomas.

#### Virtual Deletion Studies

**Figure 3:**
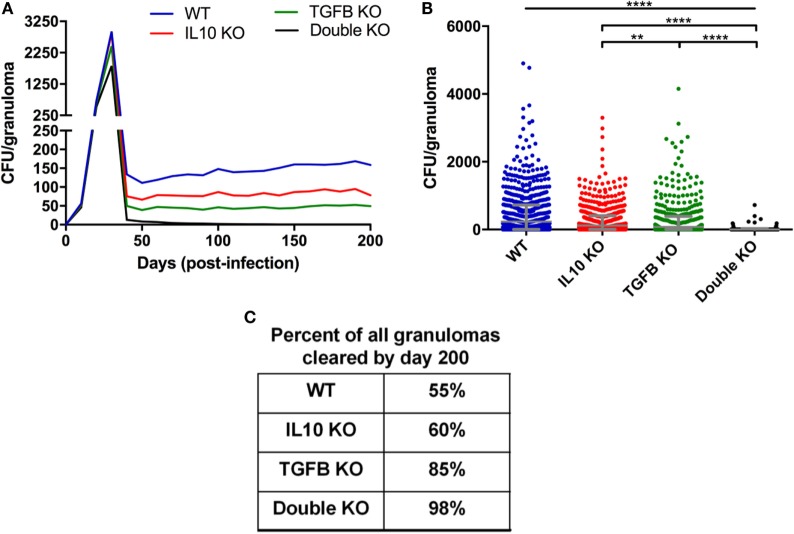
Comparison of CFU from simulated granulomas between wild-type (WT), IL-10 knockout (KO), TGF-β1 KO, and TGF-β1/IL-10 double KO. **(A)** Each line represents the mean CFU of all 1,337 simulated granulomas that did not sterilize. **(B)** Each dot represents the CFU of a single granuloma at day 200 PI. Ordinary one-way ANOVA and Sidak’s multiple comparison tests were performed to determine significance. ANOVA was highly significant with *p* < 0.0001. Sidek’s multiple comparison results are shown on the graph. ** indicates *p* < 0.01, **** indicates *p* < 0.0001. The unbarred line represents a significant difference between the WT and all KO conditions. **(C)** Table indicates percentage of simulated granulomas where CFU = 0 at day 200 PI under the aforementioned conditions.

**Figure 4:**
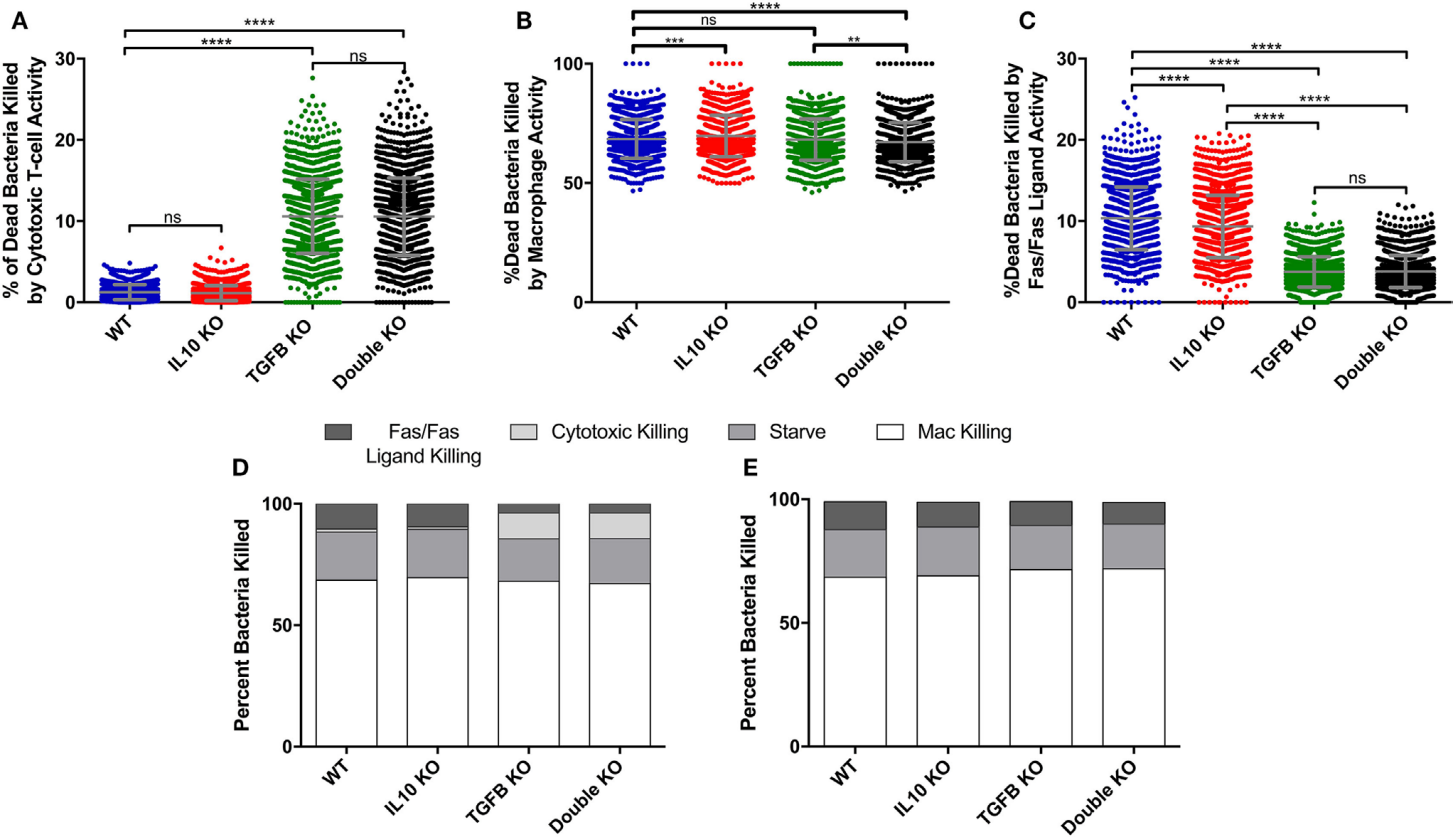
Comparison of cumulative bacterial killing by cytotoxic T-cells and macrophages between *simulated* wild-type, IL-10 knockout (KO), TGF-β1 KO, and TGF-β1/IL-10 double KO granulomas over 200 days postinfection. **(A)** Percent of dead bacteria in simulated granulomas killed by cytotoxic T-cells. Ordinary one-way ANOVA and Sidak’s multiple comparison tests were performed to determine significance. ANOVA was highly significant with *p* < 0.0001. **(B)** Percent of dead bacteria killed by macrophages in simulated granulomas. Ordinary one-way ANOVA and Sidak’s multiple comparison tests were performed to determine significance. ANOVA was highly significant with *p* < 0.0001. **(C)** Percent of dead bacteria killed by Fas/Fas-ligand in simulated granulomas. **(D)** Percent of bacterial killing caused by Fas/Fas-ligand signaling, cytotoxic effector functions, starvation, and macrophages in the four cytokine-KO sets. **(E)** Percent of bacterial killing caused by Fas/Fas-ligand signaling, cytotoxic effector functions, starvation, and macrophages in the four cytokine KO sets with the additional KO of cytotoxic T-cells. Sidek’s multiple comparison results are shown on the graph. ns (not significant) indicates *p* > 0.05, ** indicates *p* < 0.01, *** indicates *p* < 0.001, **** indicates *p* < 0.0001.

**Figure 5:**
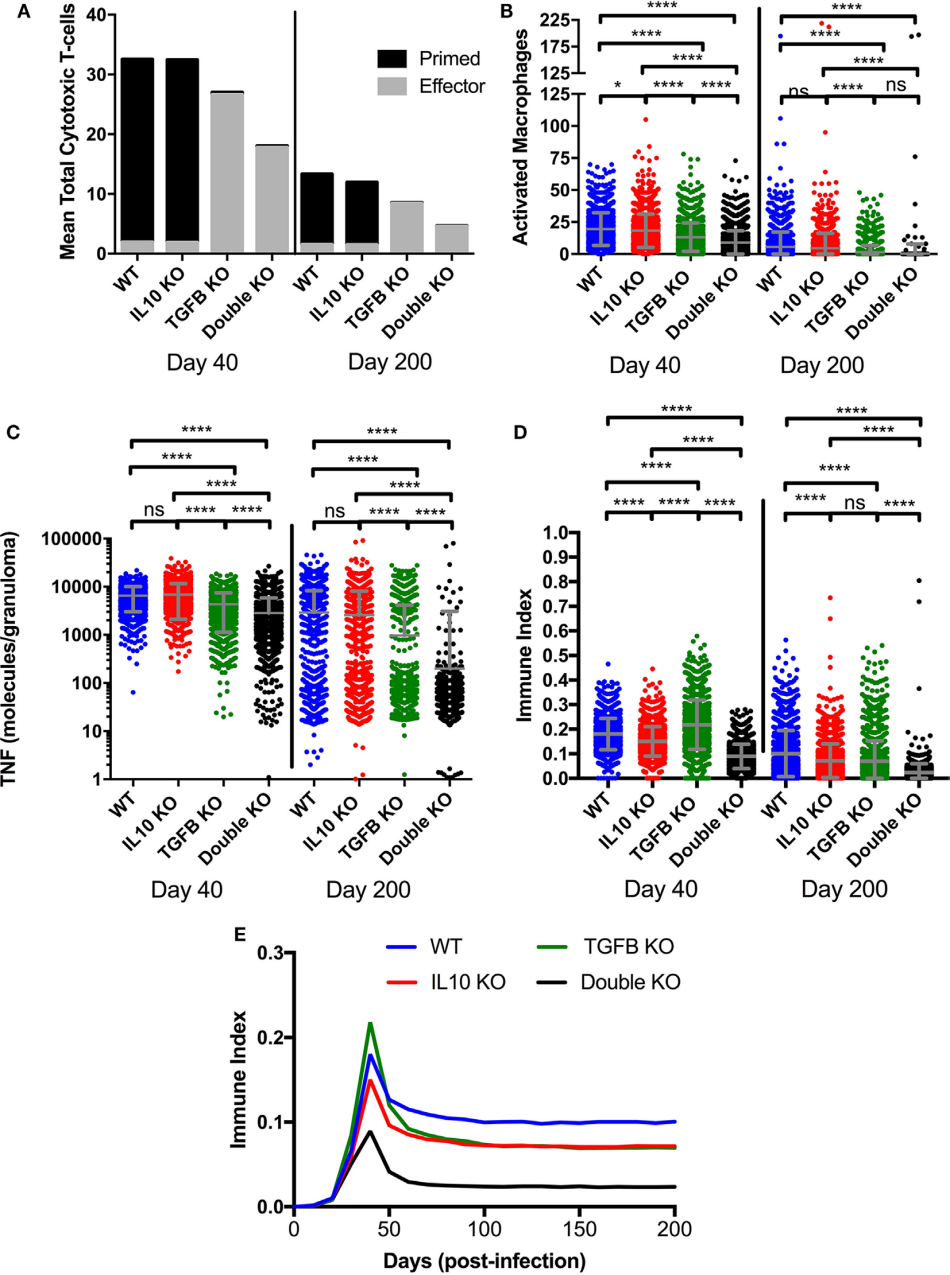
Comparison at two time points postinfection between effector and non-effector cytotoxic T-cells, activated macrophages, and total TNFα in wild-type, IL-10 knockout (KO), TGF-β1 KO, and TGF-β1/IL-10 double KO *simulated* granulomas. **(A)** Black fill indicates mean cytotoxic T-cells in 1,337 simulated granulomas. Gray fill indicates mean effector cytotoxic T-cells in 1,337 simulated granulomas. **(B)** Total number of activated macrophages per granuloma. Ordinary one-way ANOVA and Sidak’s multiple comparison tests were performed to determine significance. ANOVA was highly significant with *p* < 0.0001. **(C)** Total number of TNFα molecules per granuloma. Ordinary one-way ANOVA and Sidak’s multiple comparison tests were performed to determine significance. ANOVA was highly significant with *p* < 0.0001. Sidek’s multiple comparison results are shown on the graph. **(D)** *Immune index* of a granuloma including the summation of macrophages, IFNγ-secreting T cells, cytotoxic T cells and regulatory T cells, and TNF (see Materials and Methods). Ordinary one-way ANOVA and Sidak’s multiple comparison tests were performed to determine significance. ANOVA was highly significant with *p* < 0.0001. Sidek’s multiple comparison results are shown on the graph. ns (not significant) indicates *p* > 0.05, * indicates *p* < 0.05, **** indicates *p* < 0.0001 **(E)** Mean of immune index for granulomas within each treatment group over the course of infection.

*GranSim* uniquely enables us to perform deletions and depletions that cannot presently be performed in the NHP or other animal systems. For our virtual deletion studies, we use our baseline containment granuloma set. We re-simulate each granuloma (*N* = 1,337) in the absence of different cytokines from day 0. We simulate this by setting the initial values of specified cytokines to be 0 as well as preventing any further production of these cytokines throughout the duration of the simulation. These simulations become our virtual IL-10 knockout (KO), TGF-β1 KO, and IL-10/TGF-β1 double KO sets (Figures 3–5).

#### Measure of Inflammation

A major area of interest in our study was the possibility of change in the overall magnitude of inflammation in the presence and absence anti-inflammatory cytokines. In order to account for these possible changes, we established an *immune index*, which takes into account the total number of activated immune cells at 40 and 200 days PI (Figure 5). The immune index takes into account activated macrophages (Mac_A_), activated IFNγ-secreting T cells (Tγ_A_), activated cytotoxic T cells (Tcyt_A_), activated regulatory T cells (Treg_A_), and also includes total TNFα (TNFα). Our metric of inflammation is calculated as follows:

$$\text{ImmuneIndex}=\frac{\left(\frac{{\text{\# Mac}}_{\text{A}}}{{\text{Max \# Mac}}_{\text{A}}}+\frac{\text{\# T}\gamma_{\text{A}}}{\text{Max \# T}\gamma_{\text{A}}}+\frac{{\text{\# Tcyt}}_{\text{A}}}{{\text{Max \# Tcyt}}_{\text{A}}}+\frac{{\text{\# Treg}}_{\text{A}}}{{\text{Max \# Treg}}_{\text{A}}}+\frac{\text{TNF}\alpha }{\text{Max TNF}\alpha }\right)}{5}$$

The max # of activated macrophages refers the highest number of activated macrophages across all simulations (WT, IL10 KO, TGF-β1 KO, and double KO) at a given time point. This is consistent for each of the four cell types included in the metric.

#### Virtual Depletion Studies

**Figure 6:**
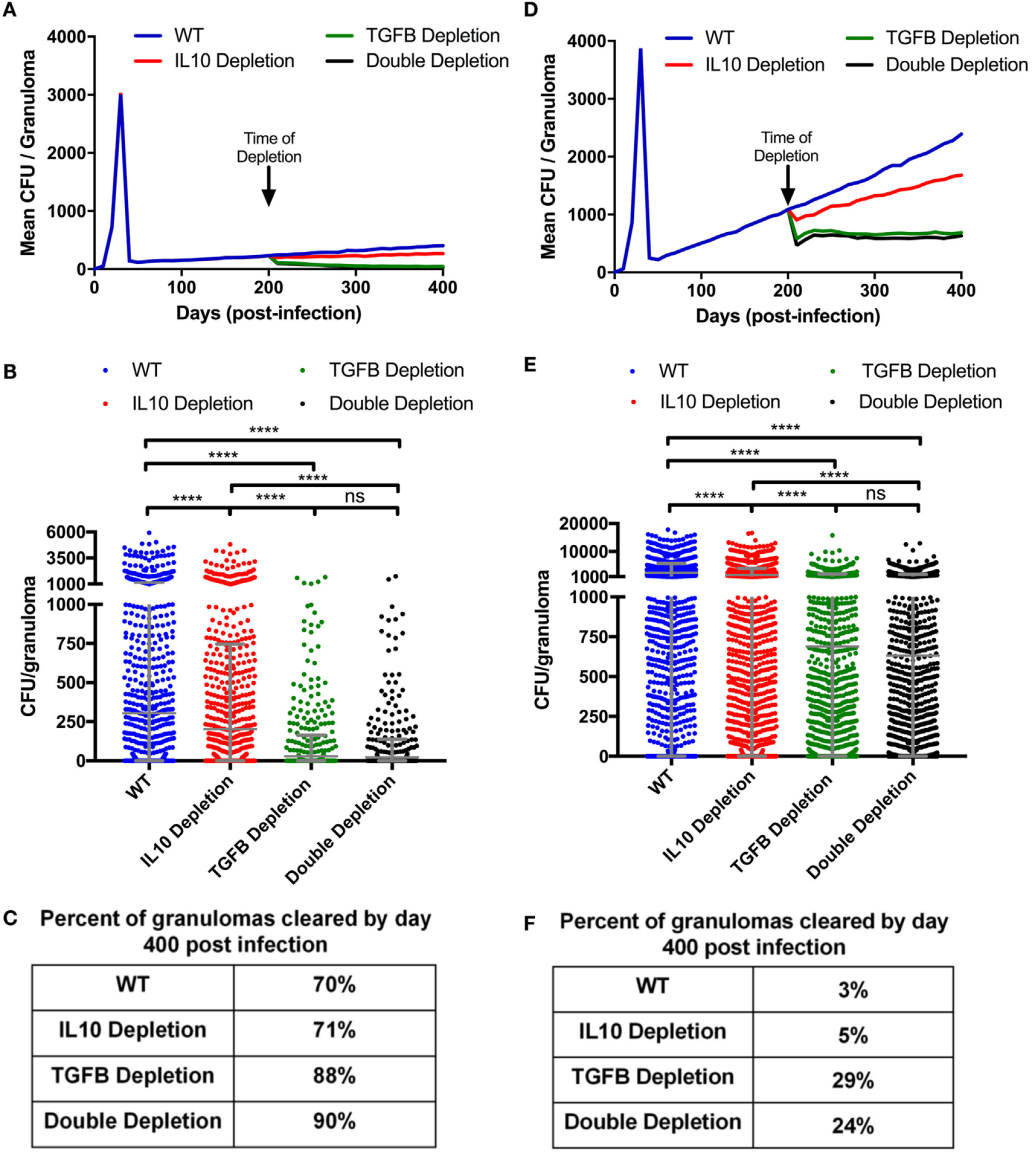
Mean CFU for cytokine depleted *simulated* granulomas at day 200 postinfection. **(A)** Mean CFU of 1,337 simulated granulomas from a containment scenario over time. **(B)** Each dot represents the CFU of a single granuloma at day 400 PI from simulations in panel **(A)**. Ordinary one-way ANOVA and Sidak’s multiple comparison tests were performed to determine significance. ANOVA was highly significant with *p* < 0.0001. **(C)** Table indicates percentage of simulated granulomas in panel **(A)** with CFU = 0 at day 400 PI. **(D)** Mean CFU of 1,337 simulated granulomas from a disseminating scenario over time with cytokine depletions at day 200. **(E)** Each dot represents the CFU of a single granuloma at day 400 PI from simulation in panel **(D)**. Ordinary one-way ANOVA and Sidak’s multiple comparison tests were performed to determine significance. ANOVA was highly significant with *p* < 0.0001. **(F)** Table indicates percentage of simulated granulomas in panel **(C)** with CFU = 0 at day 400 PI. Sidek’s multiple comparison results are shown on the graph. ns (not significant) indicates *p* > 0.05, * indicates *p* < 0.05, **** indicates *p* < 0.0001.

For our first set of virtual depletion studies, we take our virtual baseline containment set and re-simulate each granuloma (*N* = 1,337) for 200 days. At day 200 PI, we block secretion of a cytokine(s), causing concentrations in a granuloma to (rapidly) approach 0, and continue to simulate for an additional 200 days. These simulations become our IL-10 depletion, TGF-β1 depletion, and IL-10/TGF-β1 double depletion sets for contained granulomas (Figures 6A–B). We also perform depletion studies on our virtual KO dissemination set. We re-simulate the depletion set for 200 days. At day 200 PI we block secretion of different cytokines and continue to simulate for an additional 200 days. We refer to these simulations throughout the paper as our IL-10 depletion, TGF-β1 depletion, and IL-10/TGF-β1 double depletion sets for disseminating granulomas (Figures 6C–D).

#### Uncertainty and Sensitivity Analysis

Uncertainty analysis allows us to quantify how variation in parameter values drives variation in model output (79). Parameter variation can occur at the molecular and cellular scales and can affect outputs at the molecular, cellular, and tissue scales. Often variation in parameter values at one scale can influence model outcomes at another scale a phenomenon referred to as intra-model influence. Uncertainty analysis enables us to observe model behavior given a wide value range for each parameter. We vary parameters over two orders of magnitude and compare how these input variations affect model outputs. In this work, we use the Latin hypercube sampling algorithm to sample from the parameter ranges (Table S1 in Supplementary Material) and generate 500 unique parameter sets covering the full parameter space as is done in previous work (79). We use the results this uncertainty analysis to calibrate the model to *in vivo* data. We simulate each set in triplicate to account for aleatory uncertainty. We also perform uncertainty analysis where we vary only certain parameters to see how their influence on model outcomes compares. Sensitivity analyses allow us to identify which parameters have a significant influence on model outcomes and the extent of that influence (2, 79). We use partial rank correlation coefficients (PRCCs) to identify the sensitivity of each output to each parameter. PRCC values range from −1 to +1, indicating the non-linear correlation between a parameter and model output. PRCC values are differentiated using Student’s *t*-test of significance. PRCC values are considered significant with a *p*-value less than 0.01.

### Statistical Analysis

Ordinary one-way ANOVAs and Sidek’s multiple comparison tests were performed on all relevant multiple comparison data sets (85). For flow cytometry data, pairwise comparisons of receptor-positive and receptor-negative populations were performed in GraphPad Prism (La Jolla, CA, USA) with the Wilcoxon matched-pairs signed rank test.

### NHP Studies

#### Immunohistochemistry and Flow Cytometry

All samples from cynomolgus macaques (*Macaca fascicularis*) originated from animals in ongoing or completed studies performed by JoAnne Flynn at the University of Pittsburgh, and procedures and husbandry practices were included in protocols approved by the University of Pittsburgh’s Institutional Animal Use and Care Committee. Macaques were infected *via* intrabronchial instillation of virulent Mtb (Erdman strain) as previously described (13, 86). The granulomas used for flow cytometry originated from animals enrolled in vaccine studies, but there is no evidence that vaccination modifies the parameters we examined here. Flow cytometry was performed on granulomas excised from macaque lung tissue at necropsy as previously described (21). Single suspensions were prepared with either a Medimachine and Medicon system (BD Biosciences, San Jose, CA, USA) or enzymatically using a human tumor dissociation kit (Miltenyi Biotec, San Diego, CA, USA). Cells were stained for CD3 (clone SP34-2; BD Bioscience), CD11c (clone S-HCL-3; BD Bioscience), CD163 (clone GHI/61; BD Bioscience), TGFβR2 (goat polyclonal; R&D Systems, Minneapolis, MN, USA), and IL-10R (clone 3F9; Biolegend, San Diego, CA, USA) under BSL3 conditions, and fixed and stained for granzyme B (clone GB11, BD Biosciences) using the Fix-Perm reagents (BD Bioscience). Erythrocyte-free whole blood was prepared with PharmLyse RBC lysing buffer (BD Bioscience) and similarly stained as gating controls. Cells were acquired on an LSRFortessa flow cytometer (BD Bioscience) and data were analyzed with FlowJo (TreeStar Inc., Ashland, OR, USA). Gating was performed as indicated in Figure S1 in Supplementary Material and to be considered for analysis, granulomas needed to have at least 100 CD3^+^CD11c^−^ (T cells) or CD11c^+^CD163^−^ [epithelioid macrophages (5)] events. For analysis of cell surface receptor abundance, a “relative MFI” was calculated by dividing the mean fluorescence intensity (MFI) of the receptor-positive population based on the receptor-negative population’s MFI to account for the different fluorochromes and population-specific differences in autofluorescence.

Immunohistochemistry and confocal microscopy was performed on formalin-fixed paraffin-embedded granulomas as previously described (5). Serial 5 µm sections were stained with rabbit anti-human TGFβR1 (Cell Signaling Technologies, Danvers, MA, USA) or IL-10Rα (Millepore, Billerica, MA, USA), labeled with AlexaFluor546-conjugated donkey anti-rabbit secondary antibodies (ThermoFisher Scientific, Waltham, MA, USA), and coverslips were mounted with DAPI-containing Prolong Gold Mounting medium (Thermo Fisher). Isotypes for polyclonal rabbit antibodies function poorly in primate granulomas (pers. obs., J. Mattila), and so to confirm the staining with our primary antibodies, we included a serial section with rabbit anti-human phosphor-STAT3 (Cell Signaling Technologies), which stains cells in the granuloma’s lymphocyte cuff as a specificity control (Figure S1 in Supplementary Material). Individual overlapping microscopic fields were assembled into full-granuloma composites with Photoshop CS6 (Adobe, San Jose, CA, USA). For representation in the Figure 6, epithelioid macrophage-rich regions were identified by their morphologic characteristics (high cytoplasm:nucleus ratio, large lightly staining nuclei) and position adjacent to caseum (5).

## Results

### Virtual Deletion of Anti-inflammatory Mediators Decreases CFU and Improves Granuloma Sterilization

Our goal in first is to determine if anti-inflammatory mediators, specifically TGF-β1, suppress the host immune system’s ability to kill *M. tuberculosis* in the granuloma as it has been previously been suggested for IL-10 (56). We simulated a set of 1,337 contained granulomas using our *baseline parameters* (see Materials and Methods and Table S1 in Supplementary Material); these form our *wild-type (WT) containment set* of granulomas. For direct comparison, we simulated the same 1,337 granulomas three additional times in the absence of IL-10, TGF-β1, or both IL-10 and TGF-β1 to create virtual *IL-10 knockout (KO), TGF-*β*1 KO*, and *double KO* sets, respectively. Simulation results show that removal of the anti-inflammatory cytokines decreases CFU per granuloma over time (Figure 3A) with greatest decreases in the double KO set compared to WT followed by TGF-β1 KO and IL-10 KO sets, respectively. The effects of cytokine removal are visible in the double KO set as early as day 20 PI, and remain present in all simulation sets from day 30 through day 200 PI (Figure 3B). The number of lesions that sterilize increased in the absence of one or both anti-inflammatory cytokines (Figure 3C). Consistent with previously published computational studies (56), we observed that 60% of the lesions in IL-10 KO sets experienced sterilizing immunity, while TGF-β1 KO resulted in 85% of lesions becoming sterile and knocking out both IL-10 and TGF-β1 led to 98% of lesions being sterilized (Figure 3C). These simulation results suggest that TGF-β1 plays a stronger role in inhibiting bacterial clearance than IL-10.

### Cytotoxic T Cells Are Responsible for Decreased Bacterial Load in Virtual TGF-β1 KO Granulomas

To determine what cell types are mediated by TGF-β1 and IL-10 in the granuloma, we performed a sensitivity analysis on our simulations (see Materials and Methods) and identified that TGF-β1 has a strong influence on cytotoxic T cell effector function (Table S2 in Supplementary Material). Using the model, we have the ability to dissect individual activities of cells. Thus, we compared the cumulative number of bacteria killed by cytotoxic T-cell activity in our KO containment sets between day 0 and day 200 PI (Figure S2A in Supplementary Material). Granulomas without TGF-β1 killed significantly more bacteria by cytotoxic T-cell activity than any other KO set (Figure S2A in Supplementary Material). The double KO set shows significantly better bacterial killing by cytotoxic T cells than the baseline set, while the IL-10 KO sets kill significantly fewer bacteria by cytotoxic T cells (Figure S2A in Supplementary Material). We also compare sets for the total percent of bacteria killed per granuloma due to activity attributable to cytotoxic T-cells between day 0 and day 200 PI (Figure 4A). The difference in total CFU killed by cytotoxic T cells in the IL-10 KO compared WT (Figure S2A in Supplementary Material) but not in the % dead bacteria killed by cytotoxic T cells (Figure 4B) is that the IL-10 KO and the WT have different overall CFUs. The IL10 KO has overall fewer bacteria that the WT thus the same percent covers a different number of total bacteria (Figure S2A in Supplementary Material). The percent in the TGF-β1 KO and double KO sets was significantly greater than in the baseline set. There was no significant difference between the TGF-β1 KO and double KO sets. The IL-10 KO showed no significant increase in percent bacterial killing by cytotoxic T-cell activity compared to baseline granulomas. These data indicate that in the absence of TGF-β1, cytotoxic T cells kill more bacteria than they do in the presence of TGF-β1.

To confirm that this result could not be attributed to macrophage-directed behaviors, we also examined the percent and total number of bacteria killed by macrophage activity and we observed either no significant change or significant decrease in macrophage-directed behaviors between the KO containment sets (Figure 4B; Figure S2B in Supplementary Material). When we compared bacteria killed by Fas/Fas-ligand activity, we found significant reductions in the TGF-β1 KO and double KO sets compared to baseline granulomas (Figure 4C; Figure S2C in Supplementary Material). This metric refers to bacteria killed by Fas/FasL activity of macrophages and IFNγ secreting T cells, but not any killing by cytotoxic T cells. The observed decrease is attributable to lower numbers of infected macrophages over the 200 days PI in the TGF-β1 KO and double KO scenarios. In addition to killing by macrophages, cytotoxic T cells, and Fas/Fas-ligand interactions, bacteria can also be killed by starvation in the caseum of the granuloma (73). Together, these four actions account for all bacterial killing in *GranSim* (Figure 4D). We found that the effect of TGF-β1 on bacterial killing is primarily due to the effects of TGF-β1 on cytotoxic T cells (Figure 4D). In the absence of cytotoxic T cells, we observe increased bacterial killing by Fas/Fas-ligand activity that is more similar to what we see in baseline granulomas (Figure 4E). In the absence of both TGF-β1 and cytotoxic T cells, we also observed slight increases in bacterial killing by macrophages reflecting the baseline scenario (Figure 4E).

The increased bactericidal activity by cytotoxic T cells seen in the absence of TGF-β1 (Figure S2A in Supplementary Material) could be due to an increased number of cytotoxic T cells or to increased cytotoxic T cell effector functions. As shown in Figure 5A, at day 40 PI we see fewer total cytotoxic T cells in TGF-β1 KO and double KO granulomas than baseline and IL-10 KO granulomas (Figure 5A). Of the total cytotoxic T cells, approximately 6% of those in the baseline and IL-10 KO cases are effector, and approximately 98% of those in the TGF-β1 KO and double KO cases are effector (Figure 5A). The same trend occurs at day 200 PI with a mean of approximately 10% effector cytotoxic T cells in the baseline and IL-10 KO cases and a mean of approximately 98% effector cells in the TGF-β1 KO and double KO cases (Figure 5A). These results suggest that increased bacterial killing by cytotoxic T cells in the absence of TGF-β1 is due to increased cytotoxic T cell effector functions, and not an increase in numbers of cytotoxic T cells in the granuloma. We observed a decrease in the number of activated macrophages (Figure 5B) and total TNFα (Figure 5C) in the absence of TGF-β1 indicating a decrease in inflammation. Furthermore, we create an *immune index* that represents the overall immune activation levels within a granuloma. We calculate this index by summing all the activated immune cells within a granuloma including activated macrophages, activated IFNγ-secreting T cells, activated cytotoxic T cells, and activated regulatory T cells. Our immune index demonstrates that levels of inflammation increase in the absence of TGF-β1 at day 40 PI but not in the absence of both TGF-β1 and IL10 at day 40 PI (Figure 5D). Furthermore, our immune index demonstrates that levels of inflammation decrease by day 200 PI in the absence of anti-inflammatory cytokines in a granuloma (Figure 5D). This result suggests that increased inflammation is not sustained over the course of the infection simulation (Figure 5E).

### Virtual Depletion of TGF-β1 at Day 200 PI Decreases CFU and Increases Bacterial Clearance

In the simulations examined above, we focused on the early period of granuloma formation and function (0–200 days PI). To determine if TGF-β1 regulates mature granulomas, we simulated the granulomas from our baseline containment set for 400 days (Figure 6A), and then simulated the same 1,337 granulomas depleting IL-10, TGF-β1, or both IL-10 and TGF-β1 starting at day 200 PI. These simulations comprise a new group of containment sets: IL-10 depletion, TGF-β1 depletion, and double depletion sets. We observed that depleting IL-10, TGF-β1, and both IL-10 and TGF-β1 decreases the mean CFU per granuloma by day 400 PI (Figure 6B). In addition to decreased mean CFU per granuloma, we observe an increased percent of granulomas that are cleared between day 200 and day 400 PI. Simulations predicted that 70% of granulomas in our baseline containment set, and 71% in IL-10 depletion set would have sterilizing immunity by 400 days PI (Figure 6C). These results indicate that IL-10 is not playing an important role in bacterial clearance late in infection. Removing IL-10 from a fully formed contained granuloma will not increase the likelihood of bacterial clearance (56). We also predict that 88% of granulomas would clear bacteria by day 400 PI after TGF-β1 depletion (Figure 6C). This result suggests that removing TGF-β1 improves bacterial clearance in fully formed granulomas as effectively as knocking out TGF-β1 at the time of infection (Figure 3C). There was no significant difference in the effect on bacterial clearance between TGF-β1 depleted and double depleted granulomas, further demonstrating that IL-10 is not playing an important role in bacterial clearance between day 200 and day 400 PI (Figure 6C).

Taken together, these results suggest that depleting TGF-β1 in contained granulomas decreases CFU per granuloma and increases lesion sterilization. To determine if these results are consistent in disseminating granulomas, we simulated a 1,500 granuloma baseline *dissemination set*. We also simulated the same 1,500 granulomas with IL-10, TGF-β1, or both IL-10 and TGF-β1 depleted at 200 days PI. The mean CFU per granuloma of the baseline dissemination set increases continuously from day 50 PI (Figure 6D). Anti-inflammatory cytokine depletion decreases the mean CFU per granuloma (Figure 6D). The TGF-β1 and IL-10/TGF-β1 depletions show the greatest decrease in CFU compared to baseline dissemination set (Figures 6D–E). In both cases, the mean CFU per granuloma stabilizes indicating that these granulomas are no longer disseminating. The IL-10 depletion has a decreased mean CFU per granuloma relative to baseline, but continues to increase over time (Figure 6D). We also looked at the ability of disseminating granulomas to achieve sterilization under these conditions and found at 400 days PI the baseline dissemination granulomas become sterile 3% of the time, IL-10 depletion leads to sterilization in 5% of granulomas, and the TGF-β1 depletion leads to sterilizing immunity in 29% of granulomas, while the double depletion leads to sterilization in 24% of granulomas (Figure 6F). These results corroborate observations seen in the simulated KO containment granulomas (Figure 3) and further emphasize that TGF-β1 signaling inhibits granuloma sterilization.

### Macrophages and Cytotoxic T Cells Differentially Express Anti-inflammatory Cytokine Receptors in NHPs Validating Our Predictions

**Figure 7:**
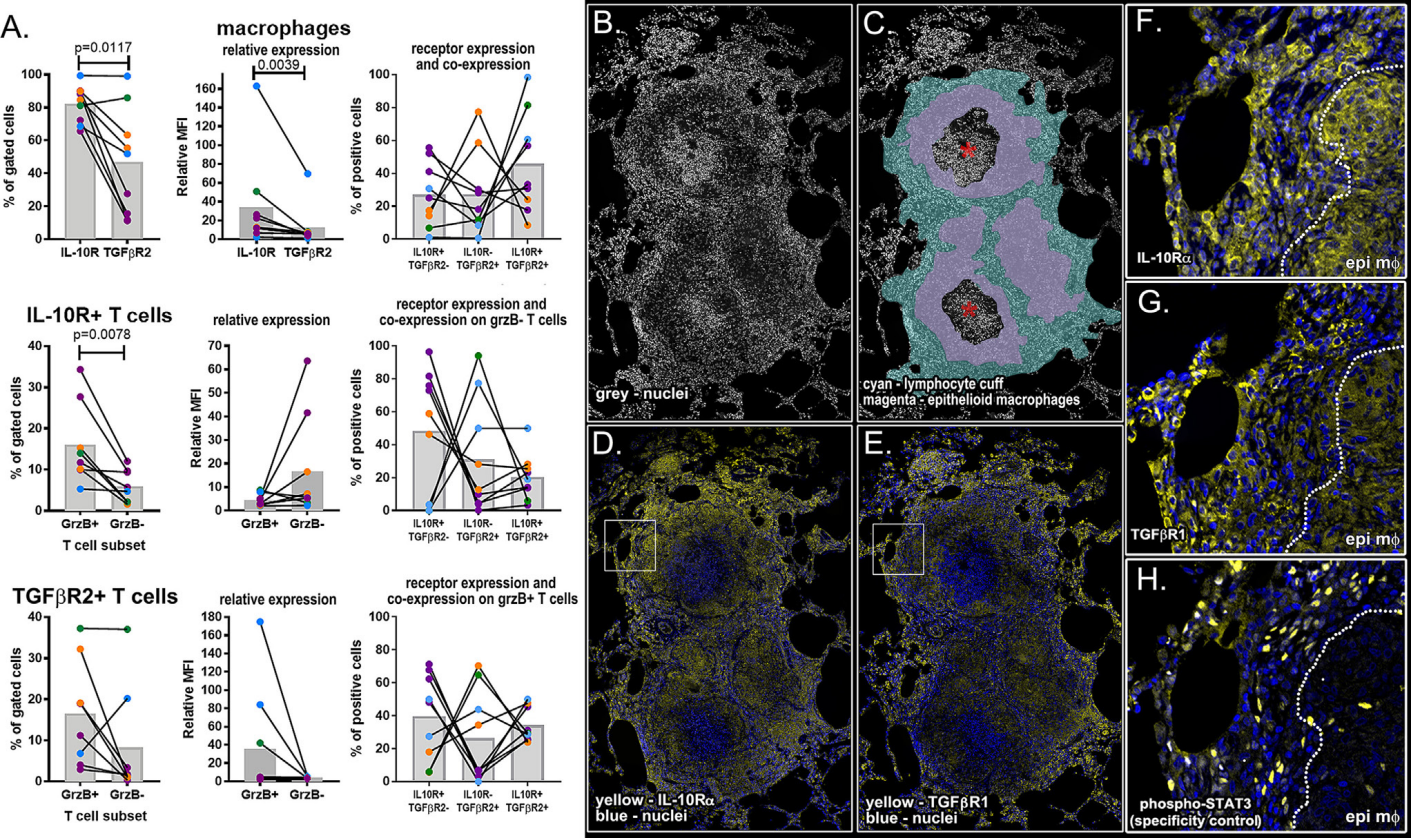
Flow cytometry and immunohistochemical analysis of IL-10 and TGFβ1 receptor for macrophages, granzyme B (grzB^+^) (cytotoxic) T cells, and grzB^−^ (non-cytotoxic) T cells. **(A)** Flow cytometry was used to compare the frequency of IL-10 and TGFβ1 receptor-positive cells (left column), relative expression per cell (center column), and Boolean gating was used to determine the frequency of receptor-positive cells expressing one or both receptors (right column). Epithelioid macrophages were defined as CD11c^+^CD163^−^ macrophages, and T cells were subdivided into cytotoxic and non-cytotoxic subsets. Each dot indicates an individual granuloma (*N* = 9) and each color indicates a different monkey (*N* = 4). Bars represent mean relative mean fluorescence intensity. **(B)** DAPI staining of necrotic granulomas showing the **(C)** cell-dense lymphocyte cuff (cyan), less dense epithelioid macrophage region (magenta), and neutrophil-rich caseum (asterisk). **(D)** IL-10R expression (yellow) is greatest in epithelioid macrophage regions [inset in panel **(F)**] while **(E)** TGFβR1 staining (yellow) is more intense in lymphocyte cuff cells [inset in panel **(G)**]. **(H)** Rabbit anti-human phospho-STAT3 was used as a specificity control.

We used a combination of flow cytometry and immunohistochemistry to compare TGF-β and IL-10 receptor expression and localization in granulomas (Figure 7). To fully characterize the biology of these receptors in a complex NHP system, we quantified the frequency of receptor-positive macrophages and examined patterns of IL-10R and TGFβR2 expression on macrophages. We performed a similar analysis for T cells, subdividing this cell population into cytotoxic (grzB^+^) and non-cytotoxic (grzB^−^) populations. Using this approach, we found larger populations of IL-10R^+^ epithelioid macrophages (Figure 7A, top left), we found higher IL-10R expression per cell relative to TGFβR2 expression (Figure 7A, top middle) although there was a substantial amount of receptor co-expression on this macrophage subset (Figure 7A, top right). We found similar frequencies of IL-10R- and TGFβR2-expressing T cells in granulomas, with a median frequency of 5.5% of IL-10R^+^ T cells (range: 0.59–31.4%) and a median of 7.5% of TGFβR2^+^ T cells (range: 0.6–38.4%) in granulomas. When comparing cytotoxic and non-cytotoxic T cells, we found significantly more IL-10R^+^ cytotoxic T cells in granulomas (Figure 7A, middle left panel), with trends toward greater expression per cell (Figure 7A, center) and greater frequencies of single-positive IL-10R^+^ cells in this subset. We found similar numbers of cytotoxic and non-cytotoxic TGFβR2^+^ T cells (Figure 7A, lower left) with a trend toward cytotoxic T cells having greater TGFβR2 expression per cell relative to non-cytotoxic T cells (Figure 7A, lower middle) although there were substantial frequencies of cytotoxic T cells that expressed both receptors (Figure 7A, lower right). We also examined IL-10R and TGF-βR1 expression in serial sections of a necrotic formalin-fixed paraffin-embedded NHP granuloma (Figure 7B) to localize receptor expression in the lymphocyte cuff and epithelioid macrophage regions (Figure 7C). We used phospho-STAT3 as a specificity control (Figure S3 in Supplementary Material, Figure 7H). Consistent with our flow cytometry data for macrophages, we found the strongest IL-10R expression corresponded with macrophage-rich regions (Figures 7D,F), while TGFβR2 expression was greatest in the lymphocyte-rich (Figures 7E,G). These data underscore the complexity of the IL-10R/TGFβR regulatory system *in vivo*, but suggest there are subtle, but quantifiable, differences between macrophage and T cell subsets that differentially impact regulation by IL-10 and TGFβ.

## Discussion

Vast numbers of people remain infected with *M. tuberculosis* despite efforts to improve diagnosis and treatment of TB. With increases in antibiotic resistance rates, and no broadly effective vaccine, novel therapeutic approaches are desperately needed to curb this pandemic. Many of the challenges in TB treatment stem from factors associated with the complex environment of granulomas. It is becoming increasingly clear that a balance of pro- and anti-inflammatory mechanisms are required for protection, and this may present opportunities for host-directed therapies for TB (5). While this strategy of manipulating the granuloma environment could improve bacterial clearance, too much of a change, changing the wrong factor, or poorly timed perturbations in either a pro- or anti-inflammatory direction could be detrimental to the host.

Combining *in silico* analyses from a multi-scale agent-based model with experimental macaque-based studies enabled us to identify previously unknown relationships between regulatory mechanisms contributing to granuloma formation and infection outcomes in TB. Our multi-scale agent-based model allows us to evaluate regulation in granulomas at multiple biological scales simultaneously over time. Because of the expedited rate of computational studies, we can simulate thousands of independent and stochastic granulomas and examine them in ways that cannot currently be accomplished *in vivo*. Our model, *GranSim*, captures many relevant biological functions and is calibrated and validated to *in vivo* data (7, 18, 19, 26, 56, 70–77, 87–93). *GranSim* does not capture the full plasticity all cell types and cytokines present in an *in vivo* granuloma. In order to address these complex biological mechanisms, without reducing the clarity of the model, function, and not surface markers, is used to characterize cells within GranSim. This way allows us to focus on the current actions of cells and not their origin. We focus on specific mechanisms described herein (see Materials and Methods) and on the GranSim website.

In this study, we identified TGF-β1-regulated mechanisms of granuloma formation, function, and bacterial control. We make assumptions regarding the interactions of TGF-β1 and immune cells based in *in vitro* and *in vivo* experimental results (see Materials and Methods). We found that TGF-β1 regulation of cytotoxic T-cell responses leads to suppression of their effector functions during infection. Our *ex vivo* studies of cytokine receptor expression and localization supports our hypothesis that TGF-β1 and IL-10 perform distinct roles in the granuloma. Our simulations suggest that depleting TGF-β1 in *M. tuberculosis*-infected granulomas can increase the effector functions of cytotoxic T cells without increasing their numbers and improve bacterial clearance without prolonged increase in inflammation. Thus, therapeutic inhibition of TGF-β1 signaling may improve lesion sterilization in TB and represent a new strategy that can be exploited to improve host responses against *M. tuberculosis*. Pirfenidone, a drug that inhibits TGF-β1 signaling, has recently been approved for treating pulmonary fibrosis (94) and our results suggest this compound may have potential to promote bacterial clearance during TB treatment. Inhibiting TGF-β1 presents clinical challenges because of its pleotropic physiologic importance, but our study highlights the importance of cytotoxic T-cell effector function in bacterial clearance and suggests stimulating cytotoxic T cells may also have therapeutic value in treating TB.

We also examined whether IL-10 and TGF-β1 represent redundant regulatory mechanisms at the site of infection and found that these cytokines differentially regulate TB granuloma macrophage and T cell subset responses. Inhibitory effects of IL-10 on macrophages have been characterized previously by our group (26, 56) and others (95). Identification of cytotoxic T-cell regulation by TGF-β1 in this context is novel, but it has been suggested in one other non-TB system that TGF-β1 predominantly regulates lymphoid-derived cells while IL-10 predominantly regulates myeloid-derived cells (65, 96). The dichotomous regulation of myeloid and lymphoid cells by anti-inflammatory cytokines has been indicated for myeloid and lymphoid cell lineages in studies outside of TB, and could help us better understand mycobacterial persistence in TB. This also has implications that span the spectrum of infectious diseases and immunological disorders. Removing TGF-β1 *in silico* improves bacterial clearance in the granuloma by enabling cytotoxic T cell effector functions without increasing potentially pathologic inflammatory responses. TGF-β1 regulation of cytotoxic T cells differs from IL-10, which has been shown to regulate macrophage activation. Identifying specific mechanisms of cytokine regulation in granulomas affords better identification of therapeutic targets for TB.

## Ethics Statement

This study was carried out in accordance with the recommendations of the University of Pittsburgh’s Institutional Animal Use and Care Committee (IACUC), which follows the guidelines established in the Animal Welfare Act and Guide for the Care and Use of Laboratory Animals (8th Edition) as mandated by US Public Health Service Policy. All animal protocols were approved by the University of Pittsburgh IACUC.

## Author Contributions

HW is the primary author of the paper who made significant intellectual contributions to the work, constructed novel components of the model, and wrote the majority of paper. EP made intellectual contributions to the work, assisted with technical challenges, and aided in review editing of the manuscript. JL made significant intellectual and advising contributions to the work, assisted with technical challenges, and aided in review editing of the manuscript. JM made intellectual contributions to the work, performed all non-human primate experiments and wrote the methods for those experiments, and aided in review editing of the manuscript. DK made significant intellectual and advising contributions to the work, assisted with technical challenges, and aided in review editing of the manuscript. DK and JL are both PIs on the grant that funded this work.

## Conflict of Interest Statement

The authors declare that the research was conducted in the absence of any commercial or financial relationships that could be construed as a potential conflict of interest.
