# Supplementary material for: Deletion of TGF-β1 Increases Bacterial Clearance by Cytotoxic T Cells in a Tuberculosis Granuloma Model
Source: Front Immunol. 2017 Dec 20;8:1843. doi: 10.3389/fimmu.2017.01843 (PMC5742530; doi:10.3389/fimmu.2017.01843)
Supplement: Supplementary file 1 [file Data_Sheet_1.docx]

**Supplemental Materials: Depletion of** **TGF-β1 Increases Bacterial Clearance by Cytotoxic T Cells in a Tuberculosis Granuloma Model**

Table S1. Parameters and parameter ranges used to generate baseline containment simulations

Table S2. Significant PRCC values for TGF-β1 parameters introduced in this version of GranSim at day 200 PI

Figure S1. Decreased secretion of TGF-β1 by macrophages results in increasing percentage of effector cytotoxic T-cells at day 200.

Figure S2. Gating strategy for flow cytometry studies.

Table S1. Parameters and ranges used to generate baseline containment simulations

| **Parameter name** | **Value or range** | **Units** |
| --- | --- | --- |
| **Mtb parameters** | | |
| Growth rate intracellular Mtb | 1.003 | cells |
| Growth rate extracellular Mtb | 1.001 | cells |
| Death rate of extracellular Mtb in caseum | 1.5 | cells |
| **Core model parameters** | | |
| Diffusion time step | 60 | seconds |
| Molecular time step | 6 | seconds |
| Diffusion smoother time step | 1.2 | seconds |
| Number of smoother steps | 0 | n/a |
| Number of host cells causing caseation | 10 | n/a |
| Time to heal caseation | [1642, 2462] | days |
| Threshold for TNFα induced apoptosis | [1393, 2089] | molecules |
| Rate of TNFα induced apoptosis | [1.17e-6, 1.76e-6] | 1/seconds |
| Minimum number of molecules allowing chemotaxis | [0.514, 0.77] | molecules |
| Maximum number of molecules allowing chemotaxis | [374, 562] | molecules |
| Diffusivity of TNFα | 5.2e-8 | cm^2^/s |
| Diffusivity of IL10 | 5.2e-8 | cm^2^/s |
| Diffusivity of active TGF-β1 | 5.2e-8 | cm^2^/s |
| Diffusivity of chemokines | 5.2e-8 | cm^2^/s |
| Degradation rate of TNFα | 0.00158 | molecules/molecular time step |
| Degradation rate of IL10 | 0.00048 | molecules/molecular time step |
| Degradation rate of inactive TGF-β1 | [9.28e-6, 1.39e-5] | molecules/molecular time step |
| Degradation rate of active TGF-β1 | [8.0e-4, 0.0012] | molecules/molecular time step |
| **Macrophage parameters** | | |
| Fraction of grid compartments with a macrophage | [0.024, 0.036] | n/a |
| Number of time steps before a resting macrophage can move | 2 | n/a |
| Number of time steps before an activated macrophage can move | 16 | n/a |
| Number of time steps before an infected macrophage can move | [112, 168] | n/a |
| Synthesis rate of TNFα  (${MacTNF\alpha}_{synth}$) | 1.5 | molecules/diffusion time step |
| Synthesis rate of CCL2 | 6 | molecules/diffusion time step |
| Synthesis rate of CCL5 | 6 | molecules/diffusion time step |
| Synthesis rate of CCL9 | 12 | molecules/diffusion time step |
| Synthesis rate of IL10 by an activated macrophage | 0.3 | molecules/diffusion time step |
| Synthesis rate of IL10 by and infected macrophage | 0.02 | molecules/diffusion time step |
| Synthesis rate of Inactive TGF-β1 by macrophages | [1.4e-4, 2.12e-4] | molecules/diffusion time step |
| Number of bacteria a resting macrophage can phagocytose | 1 | n/a |
| Probability of resting macrophage killing bacteria  (${MacKill}_{baseline}$) | [0.23, 0.35] | n/a |
| Threshold for intracellular bacteria causing chronically infected macrophages | [8,12] | bacteria |
| Threshold for intracellular bacteria causing macrophage to burst | [13, 20] | bacteria |
| Number of bacteria an activated macrophage can phagocytose | [4, 6] | n/a |
| Fraction of inactive TGF-β1 activated by a mac  (${Activation}_{fraction})$ | [7e-5, 1e-4] | n/a |
| Amount of TGF-β1 that inhibits macrophages  (${TGF\beta1max}_{Mac}$) | [0.01, 1] | molecules |
| Fraction of active TGF-β1 in a compartment bound by a mac | [1.1e-5, 1.7e-5] | n/a |
| Probability of an activated macrophage healing a caseated compartment in its Moore neighborhood | [0.0128, 0.0129] | n/a |
| **T cell parameters** | | |
| Probability of a T cell moving to the same compartment as a macrophage | [0.05, 0.08] | n/a |
| Probability of a T cell moving to the same compartment as a T cell | 0.08 | n/a |
| Synthesis rate of TNFα by IFNγ-producing T-cell | 0.15 | molecules/diffusion time step |
| IFNγ-producing T-cell probability of inducing Fas/FasL mediated apoptosis | [0.0152, 0.0228] | n/a |
| Probability of IFNγ-producing T-cell to secrete TNFα | [0.048, 0.072] | n/a |
| Probability of IFNγ-producing T-cell to secrete TNFα | [0.288, 0.432] | n/a |
| Synthesis rate of TNFα by cytotoxic T-cell | 0.015 | molecules/diffusion time step |
| Probability of a cytotoxic T-cell killing a macrophage | [0.012, 0.18] | n/a |
| Probability of a cytotoxic T-cell killing a macrophage and all associated Mtb | [0.61, 0.91] | n/a |
| Probability of cytotoxic T-cell to secrete TNFα | [0.056, 0.084] | n/a |
| Synthesis rate of IL10 by regulatory T-cell | 0.739 | molecules/diffusion time step |
| Synthesis rate of TGF-β1 by regulatory T cell | [0.0067, 0.0101] | molecules/diffusion time step |
| Probability a regulatory T cell will deactivate an activated macrophage | [0.011, 0.016] | n/a |
| Amount of TGF-β1 that inhibits T cells  (${TGF\beta1max}_{Tcell}$) | [0.01, 0.1] | molecules |
| **Recruitment Parameters** | | |
| Maximum macrophage recruitment probability | [0.112, 0.168] | n/a |
| Maximum IFNγ-producing  T cell recruitment probability | [0.112, 0.168] | n/a |
| Maximum cytotoxic T cell recruitment probability | [0.079, 0.12] | n/a |
| Maximum regulatory T cell recruitment probability | [0.0232, 0.0348] | n/a |

* Indicates estimated parameters. All other parameters derived from prior work (70).

Table S2: Significant PRCC values for TGF-β1 parameters introduced to *GranSim* at day 200 PI. p > 0.001

| Model outputs of interest | | Parameters | | | | | | | |
| --- | --- | --- | --- | --- | --- | --- | --- | --- | --- |
|  |  | Degradation Rate of Active  TGF-β1 | Degradation Rate of Inactive TGF-β1 | Synthesis of Inactive TGF-β1 by Macs | Fraction of  TGF-β1 Activated by Macs | Macrophage TGF-β1 Binding Rate | Maximum TGF-β1 Bound by a T cell | TGF-β1 Inhibition of Cytotoxic T cells | Synthesis of Inactive TGF-β1 by Regulatory T Cells |
| Outputs | # Total Macs |  |  |  |  |  |  | 0.09 |  |
|  | # Resting Macs |  |  |  |  |  |  | 0.08 |  |
|  | # Infected Macs |  | 0.08 |  |  |  |  | 0.10 |  |
|  | # Chronically Infected Macs |  |  |  |  |  |  |  |  |
|  | # Activated Macs |  | 0.07 |  |  |  |  | 0.08 |  |
|  | # Dead Macs |  |  |  | -0.09 |  |  |  |  |
|  | # Total IFNg+  T cells |  |  |  |  |  |  | 0.08 |  |
|  | # Active IFNg+ T cells |  |  |  |  |  |  | 0.08 |  |
|  | # Down Regulated IFNg+ T cells |  |  |  |  |  |  |  |  |
|  | # Dead IFNg+  T cells |  |  |  |  |  |  |  |  |
|  | # Total Cytotoxic  T cells |  |  |  |  |  |  | 0.09 |  |
|  | # Effector Cytotoxic  T cells |  | 0.09 |  |  |  |  |  |  |
|  | # Total Regulatory  T cells |  |  |  |  |  |  | 0.07 |  |
|  | # Intracellular  Mtb |  | 0.08 |  |  |  |  | 0.09 |  |
|  | # Extracellular Mtb |  |  |  |  |  |  | 0.09 |  |
|  | # Non-replicating Extracellular Mtb |  |  |  |  |  |  | 0.09 |  |
|  | Total CFU |  | 0.08 |  |  |  |  | 0.10 |  |
|  | Total CEQ |  | 0.09 |  |  |  |  | 0.16 |  |
|  | # Mtb  Killed by Apoptosis |  | 0.09 |  |  |  |  | 0.17 |  |
|  | # Mtb Killed by Cytotoxicity |  | 0.08 |  |  |  |  | 0.13 |  |
|  | # Mtb Killed by Fas/Fas-ligand |  | 0.08 |  |  |  |  | 0.16 |  |
|  | # Mtb Killed by Macs |  | 0.09 |  |  |  |  | 0.13 |  |
|  | # Mtb Killed in Caseation |  |  |  |  |  |  | 0.14 |  |
|  | Total TAG |  | 0.08 |  |  |  |  | 0.14 |  |
|  | Total TNF |  |  |  |  |  |  | 0.08 |  |
|  | Total CCL2 |  |  |  |  |  |  | 0.08 |  |
|  | Total CCL5 |  |  |  |  |  |  | 0.10 |  |
|  | Total CXCL9 |  |  |  |  |  |  | 0.10 |  |
|  | | Parameters | | | | | | | |
|  |  | Degradation Rate of Active  TGF-β1 | Degradation Rate of Inactive TGF-β1 | Synthesis of Inactive TGF-β1 by Macs | Fraction of  TGF-β1 Activated by Macs | Macrophage TGF-β1 Binding Rate | Maximum TGF-β1 Bound by a T cell | TGF-β1 Inhibition of Cytotoxic T cells | Synthesis of Inactive TGF-β1 by Regulatory T Cells |
| Outputs | Total TAG |  |  |  |  |  |  | 0.10 |  |
|  | Total Active TGFB |  |  | 0.07 |  |  |  | 0.09 |  |
|  | Total Inactive TGFB |  | -0.28 | 0.23 |  |  |  |  | 0.15 |
|  | Total TNFα Induced Mac Apoptosis |  | 0.08 |  |  |  |  | 0.13 |  |
|  | TNFα Induced Resting Mac Apoptosis |  | 0.07 |  |  |  |  | 0.11 |  |
|  | TNFα Induced Infected Mac Apoptosis |  | 0.09 |  |  |  |  | 0.14 |  |
|  | Total Chronically Infected Mac Apoptosis |  |  |  |  |  |  | 0.19 |  |
|  | TNFα Induced Activated Mac Apoptosis |  | 0.07 |  |  |  |  | 0.12 |  |
|  | TNFα Induced  T cell Apoptosis |  |  |  |  |  |  | 0.11 |  |
|  | Fas/FasL Killing |  | 0.08 |  |  |  |  | 0.15 |  |
|  | Cytotoxic Killing |  |  |  |  |  |  | 0.12 |  |
|  | Total Chronically Infected Mac Bursting |  | 0.10 |  |  |  |  | 0.15 |  |
|  | Total Pet Hot |  |  |  |  |  |  | 0.08 |  |

Macrophage TGFB binding rate, T cell TGFB binding rate had no significant correlations at day 200 PI*


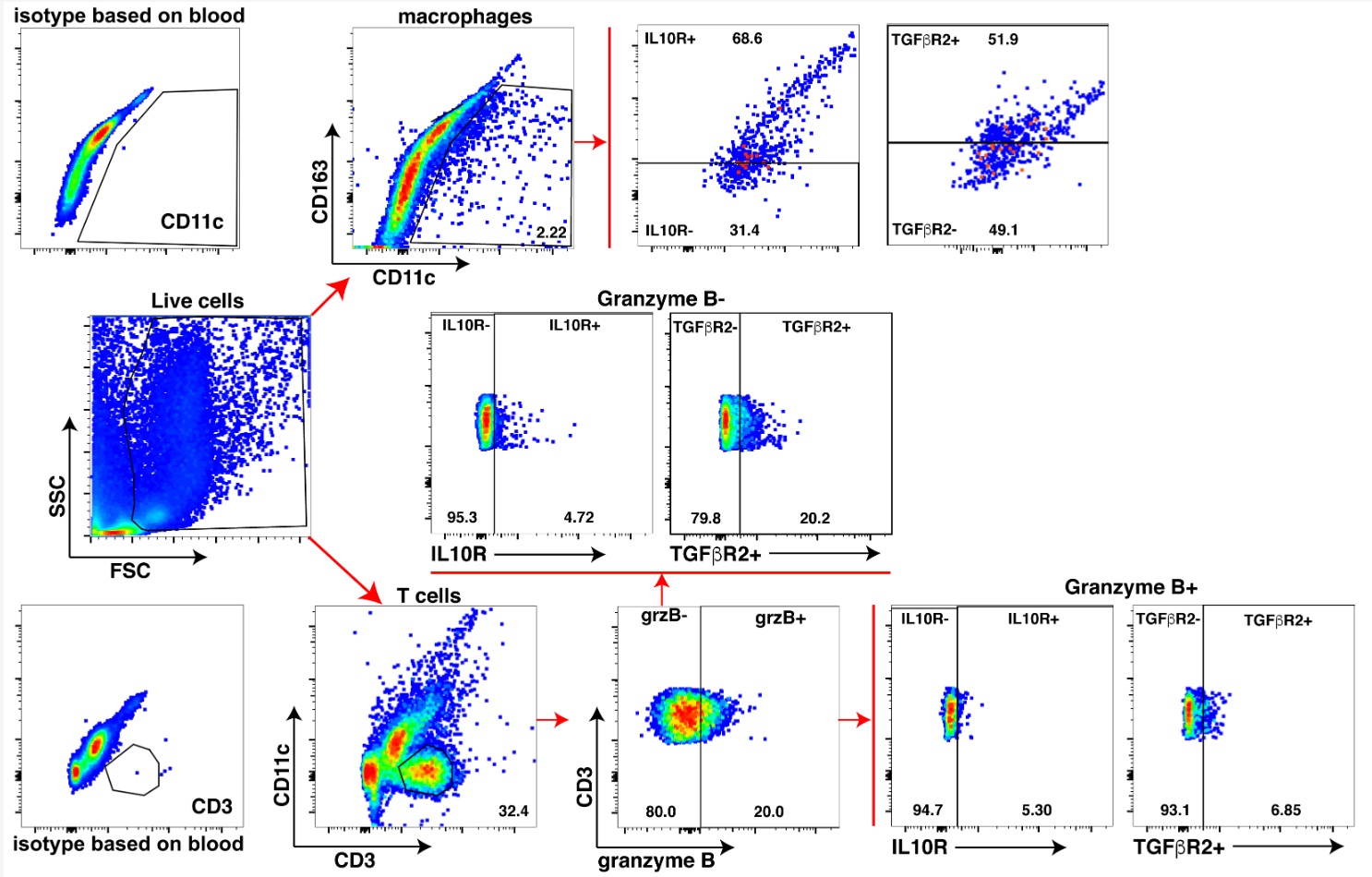


**Figure S1: Gating strategy for flow cytometry studies.** Granulomas do not contain enough cells for gating controls, and so positive and negative gates were determined by gating on erythrocyte-lysed whole blood (not shown) and these gates were then applied to granuloma samples. Isolated granuloma cells were gated on viable cells to exclude small, low complexity objects that confound analysis and then macrophages and T cells were identified by surface marker expression. Epithelioid macrophages and T cells were identified as CD11c+CD163- and CD3+CD11c- cells, respectively. T cells underwent a second round of gating against granzyme B to differentiate cytotoxic (granzyme B+) and noncytotoxic (granzyme B-) T cells. Subsequent analysis was done by gating each population’s primary surface marker (CD11c or CD3) against IL10R or TGFBR2 expression, and comparing the MFI of positive and negative populations.


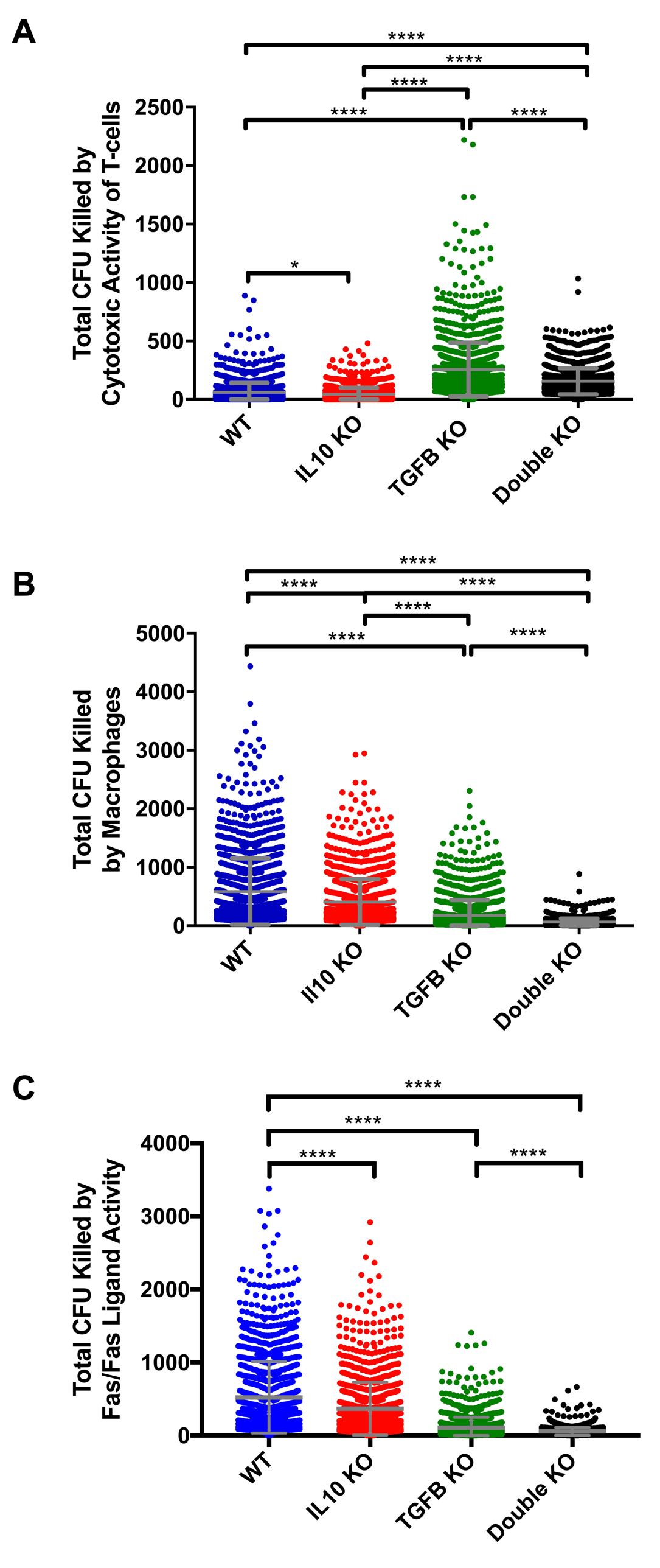


**Figure S2: Comparison of cumulative bacterial killing by cytotoxic T-cells and macrophages between *simulated* wild type, IL-10 knockout (KO), TGF-β1 knockout, and TGF-β1/IL-10 double knockout granulomas over 200 days post-infection.** A) CFU per granuloma killed by cytotoxic T-cells in 1337 simulated granulomas. Ordinary one-way ANOVA and Sidak’s multiple comparison tests were preformed to determine significance. ANOVA was highly significant with p<0.0001. B) CFU per granuloma killed by macrophages in simulated granulomas. Ordinary one-way ANOVA and Sidak’s multiple comparison tests were preformed to determine significance. ANOVA was highly significant with p<0.0001. C) CFU per granuloma killed by Fas/Fas-ligand in simulated granulomas. Ordinary one-way ANOVA and Sidak’s multiple comparison tests were preformed to determine significance. ANOVA was highly significant with p<0.0001. F) Percent of dead bacteria killed by Fas/Fas-ligand in simulated granulomas. Sidek’s multiple comparison results are shown on the graph. ns (not significant) indicates p > 0.05, * indicates p < 0.05, ** indicates p < 0.01, *** indicates p < 0.001, **** indicates p < 0.0001


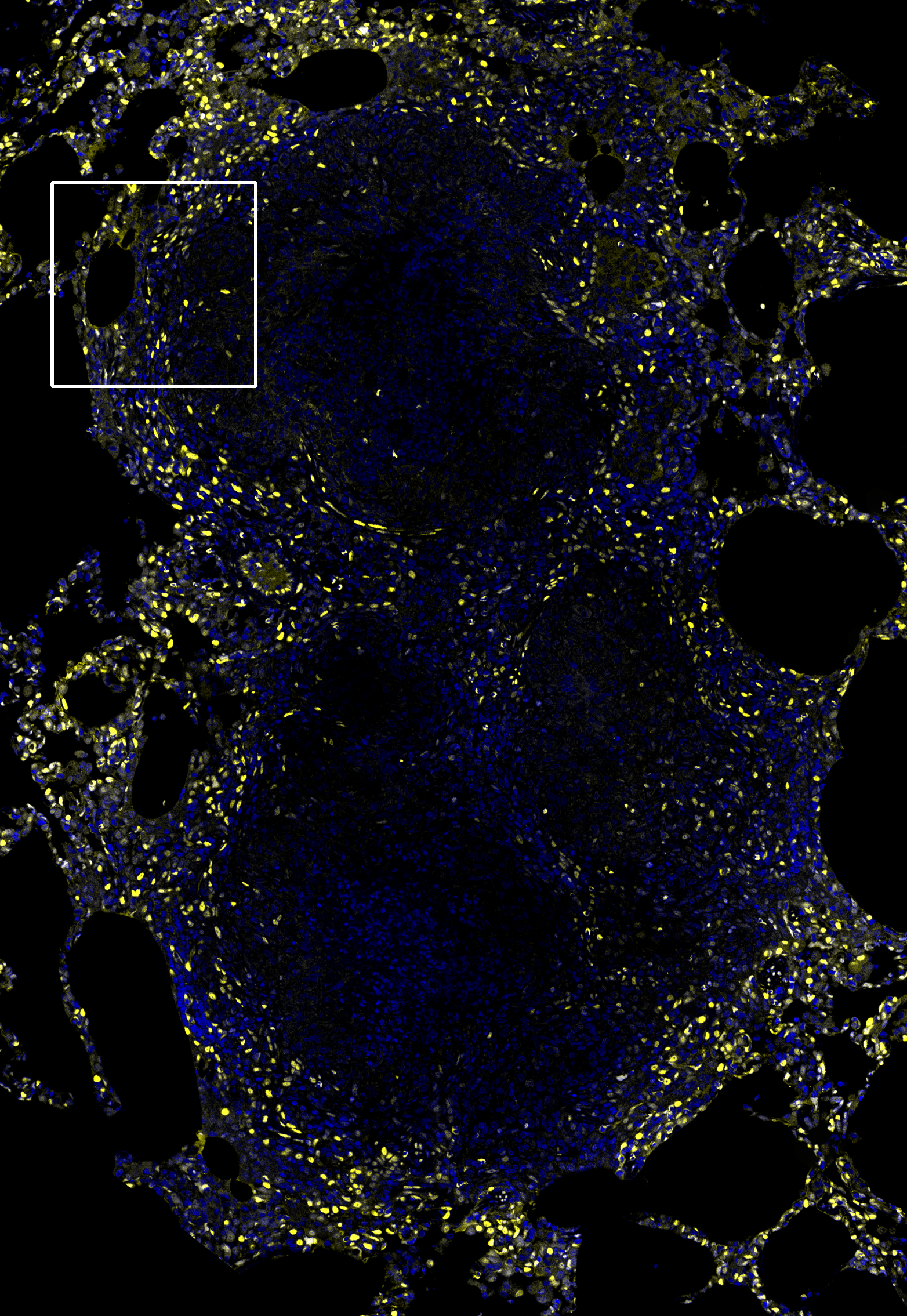


**Figure S3. Phospho-STAT3 expression (yellow) as a specificity control for anti-IL-10Ra and TGF-BR1 staining.** Inset area is shown in Fig. 6H.

**
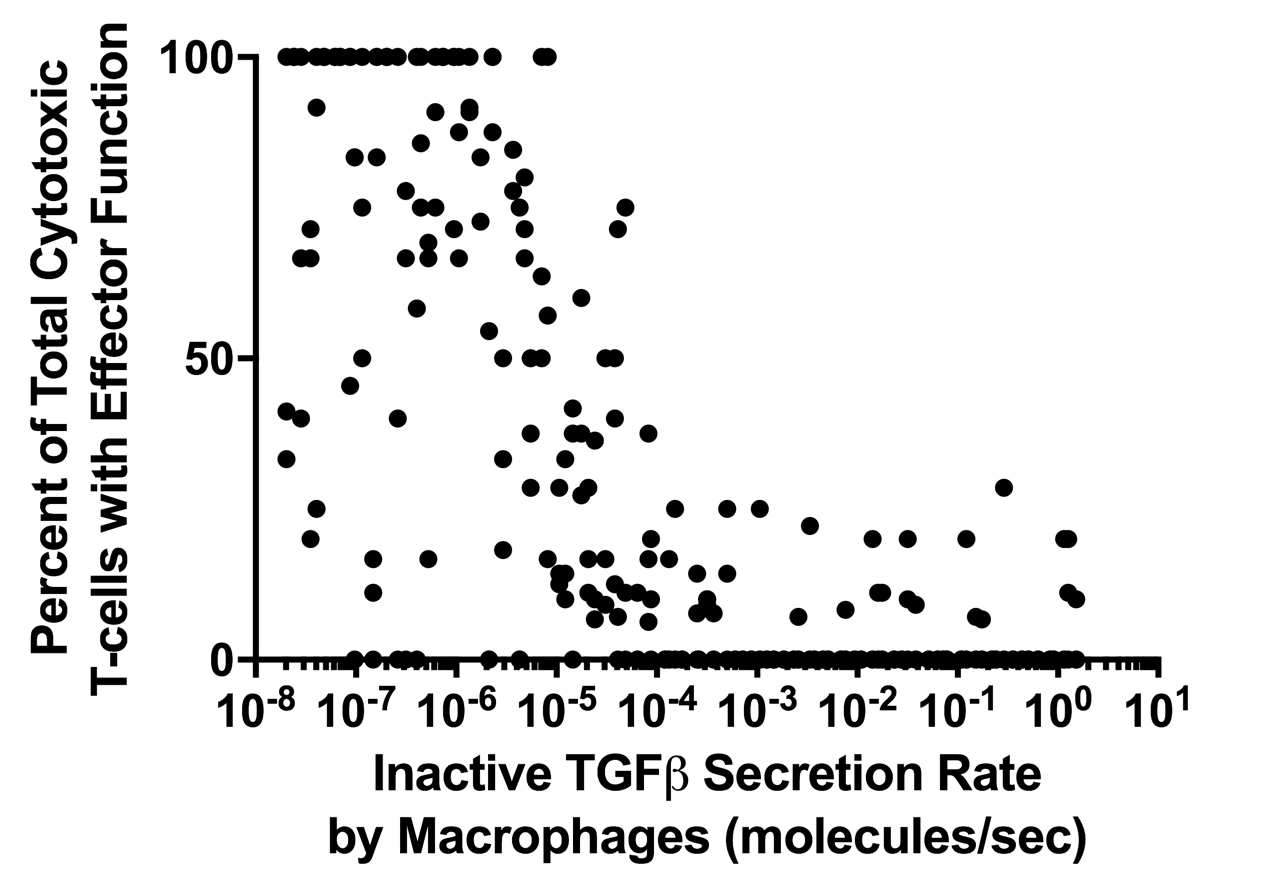
**

**Figure S4: Simulated decreased secretion of TGFβ by macrophages results in increasing percentage of effector cytotoxic T-cells at day 200.** 300 granulomas were simulated for 200 days with differing rates of latent TGFβ secretion by macrophages. The rate of secretion by macrophages is plotted against percent of total cytotoxic T-cells that are effector cytotoxic T-cells at day 200.

Effector cytotoxic T-cell activity shows sensitivity to different rates of TGF-β1 secretion.

Cytotoxic T-cell effector activity, and therefore bacterial killing efficiency, is inhibited by TGF-β1 signaling. In the absence of TGF-β1, effector cytotoxic T-cells in the granuloma are increased in number. Since macrophages are a major contributor to TGF-β1 levels in the granuloma, we compare how TGF-β1 secretion rates by macrophages affects the percent of effector cytotoxic T cells that are in granulomas (Fig. S1). We predict there is a negative correlation between the secretion rate of TGF-β1 and the percent of effector cytotoxic T cells in the granuloma (Fig. S1). In order to see a meaningful increase in the effector functions of cytotoxic T cells in our simulations, the TGF-β1 secretion rate by macrophages required a decrease by several orders of magnitude (Fig. S1).
